# The Challenge of Endoleaks in Endovascular Aneurysm Repair (EVAR): A Review of Their Types and Management

**DOI:** 10.7759/cureus.39775

**Published:** 2023-05-31

**Authors:** Vishnu R Yanamaladoddi, Sai Suseel Sarvepalli, Shree Laya Vemula, Saikumar Aramadaka, Raam Mannam, Rajagopal Sankara Narayanan, Arpit Bansal

**Affiliations:** 1 Research, Narayana Medical College, Nellore, IND; 2 Research, ACSR Government Medical College, Nellore, IND

**Keywords:** aaa repair, ruptured aaa, endograft implantation, minimally invasive surgery, endoleak repair, aaa - abdominal aortic aneursym, endovascular aortic repair (evar)

## Abstract

An abdominal aortic aneurysm (AAA) is a dilatation of the abdominal aorta above 3 cm or 50% greater than the segment above. It is a dangerous condition accounting for a substantial number of deaths per year and increasing at an alarming rate. Various factors come into play in the development of AAAs, which this study has elaborated on, including smoking and old age, demographics, and comorbid conditions. Endovascular aneurysm repair (EVAR) is a newer treatment modality used for AAAs in which an endograft device is placed into the aorta, thereby creating a bypass tract from the aneurysm and generating flow mimicking that of the natural aorta. It is minimally invasive and associated with less postoperative mortality and reduced hospital stay. However, EVAR is also associated with significant postoperative complications, including endoleaks, which were reviewed in depth. Endoleaks are postprocedural leaks into the aneurysm sac that are usually identified immediately after graft placement and indicate treatment failure. They are of five subtypes, categorized according to their mechanism of development. The most common type is type II endoleaks, and the most dangerous is type I endoleaks. Each subtype has multiple management options with varying rates of success. Prompt identification along with appropriate treatment of endoleaks can lead to better postoperative outcomes and improved quality of life for patients.

## Introduction and background

An abdominal aortic aneurysm (AAA) is a dangerous condition with vague or no symptoms and sudden rupture, leading to death in up to 70% of patients [1]. Early detection followed by endovascular aneurysm repair (EVAR) has been shown to give the best outcome in terms of survival for patients [2]. According to the Society for Vascular Surgery, an AAA is defined as an enlargement of the aorta below the diaphragm, which is greater than 30 mm in diameter or 50% greater than the part above [3]. Before the discovery of stent-graft technology, open surgery was the gold standard treatment for AAA repairs [4]. The publication of Juan Carlos Parodi's paper in 1991 on minimally invasive EVAR, along with subsequent studies highlighting benefits such as reduced general anesthesia time, elimination of pain, shortened hospital stays, and minimal blood loss, contributed to the widespread adoption of EVAR as a prevalent treatment for AAAs [5].

**Figure 1:**
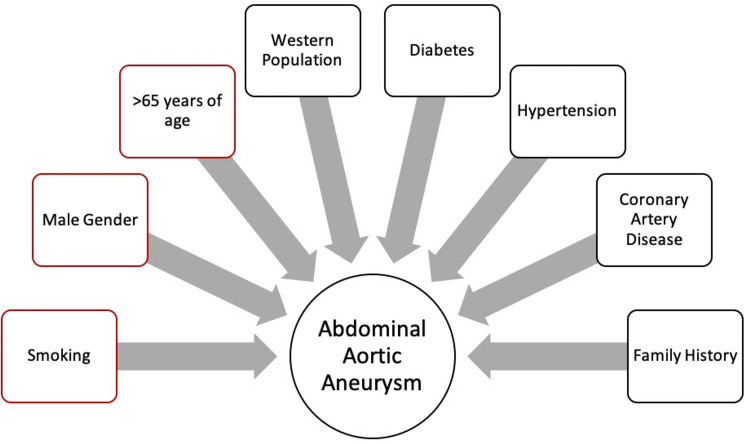
Risk factors associated with the development of AAAs. Sources: [7-13]. AAA, abdominal aortic aneurysm

Annually, the total number of deaths attributed to aortic aneurysms has been estimated to be nearly 173,000, increasing by nearly 82.1% since the discovery of EVAR [6]. Globally, the prevalence of AAAs is 4.8%. AAAs are nearly four times more predominant in men as compared to women. Age preponderance has been found mainly in the 65 and above age group [7]. The risk of AAA was found to be more in the White and Native American populations as compared to Black, Hispanic, and Asian groups [8]. Modifiable factors include smoking, hypertension, diabetes mellitus, and coronary artery disease [9] along with excess weight and a healthy diet [8]. Nonmodifiable factors include male gender and prior family history. Smoking is the most dangerous risk factor associated with AAAs. The risk for rupture is increased in patients who have a decreased forced expiratory volume in 1 second (FEV1), patients with a higher mean blood pressure, female patients [10], transplant patients [11], patients with a larger AAA diameter [12], and chronic smokers [13] (Figure 1).

The abdominal aorta is an elastic artery, which is believed to be more prone to degeneration due to being avascular and having fewer layers as compared to the rest of the aorta [14]. Aneurysms develop due to the fragmentation and degradation of the elastin and collagen in the wall of the aorta, dependent on the balance between matrix metalloproteins (MMPs) and tissue inhibitors on metalloproteinases (TIMPs), which facilitate the degradation, along with leucocyte infiltration [15].

AAAs have different presentations depending on whether they are intact or ruptured. Unruptured AAAs are mostly asymptomatic and discovered incidentally on examination or may be diagnosed after the occurrence of complications such as thrombosis and embolization. Intact aneurysms may expand and exert pressure on adjacent anatomical structures, leading to vague pain in and around the abdomen and back. Ruptured aneurysms present with a classic triad of sudden onset abdominal/flank pain, a pulsatile abdominal mass, and shock. Anterolateral wall ruptures are often fatal, while posterolateral wall ruptures may be insidious in onset and progress to a larger fatal rupture [15].

AAAs are initially diagnosed via ultrasonography (USG), which can also help measure the size and orientation of the aneurysm with an accuracy of 3 mm [16]. A computed tomography (CT) scan with contrast is the gold standard to help determine the management modality. Extravasation of contrast into the body is indicative of a ruptured AAA [15]. Recent advances in the diagnosis of AAAs include the use of magnetic resonance angiography (MRA), where there is a three-dimensional reconstruction of the patient's blood vessels [17], and CT-USG fusion technology where there is synchronization and overlay CT and USG images to enable accurate targeting and real-time guidance of the aorta [18], both of which have shown promising results in more accurate diagnosis of AAAs.

The management of an AAA depends on a multitude of factors and the treatment protocol is arranged according to the National Institute for Health and Care Excellence (NICE) Guidelines [19].

EVAR is a fluoroscopic image-guided surgical procedure in which an endoprosthesis, also known as a stent graft, is introduced into the abdominal aorta. The endoprosthesis is made of a self-expanding metal frame and a fabric wall. The stent graft is then secured both proximally and distally to the dilated part of the aorta and then sealed against the aortic wall using radial force, and a new pathway for blood flow is created by bypassing the saccular dilatation, effectively creating a new vascular wall.

While endovascular repair of aneurysms has transformed the way AAAs are treated, it is essential to discuss the complications associated with the surgery to understand the root cause of the complications and how they can be avoided. EVAR may present with a series of postoperative complications due to poor selection of patients for surgery or improper surgical technique. These include implant rejection, occlusion, pelvic ischemia, limb thrombosis, endoleak, graft migration, and sac rupture. Patients are also at risk for radiation-related complications. Endoleaks are the most crucial complications following EVAR and the most avoidable [20].

This study aims to elaborate further on endoleaks associated with EVAR and explore their various treatment modalities.

## Review

Procedure

EVAR is performed under general anesthesia. Access to the common femoral artery is obtained via skin incision or percutaneous arterial puncture on both limbs, creating an ipsilateral and contralateral internal iliac artery access. The ipsilateral artery is used to introduce the main body of the device, and the contralateral artery is used to introduce the contralateral iliac limb. The ipsilateral artery is selected so that it is ideal to work with to minimize complications, and it is preferred to be of large diameter and without stenosis or tortuosity. A calibrated pigtail catheter is introduced into the contralateral artery, and a preliminary digital subtraction arteriogram is taken to confirm the location of the renal arteries and ipsilateral internal iliac artery and determine the length of the endovascular device required. A stiff guidewire is introduced into the ipsilateral artery, and the main body device is delivered. It is placed with the fabric part covering the body of the aneurysm and distal to the lowest renal artery. Using another guide wire in the contralateral artery, an opening in the main body, called the gate, is cannulated. The gate is the link to attach the contralateral shorter limb. Another angiogram is performed to identify the length of the contralateral limb. A stiff wire is introduced and advanced to the contralateral gate, and the limb is deployed. The point of overlap between the main body and the contralateral limb is expanded using a low-pressure balloon. Angiography is done to ensure the accuracy of graft placement along with proper seal and patency. Patients are under intensive care at the hospital for three to five days and discharged with medical therapy [20].

The postoperative management of EVAR repair involves medical treatment and imaging surveillance. Medical therapy is aimed at patients at risk for or with preexisting peripheral artery disease. The Society for Vascular Surgery recommends surveillance with a one-month contrast CT scan. If there are any concerning findings, including sac enlargement and endoleaks, further surveillance should be conducted via contrast CT scan at six and 12 months and yearly thereafter. However, if no concerning findings exist, a color duplex ultrasound can be used annually to screen for complications. Ultrasound eliminates radiation exposure as well as toxicity associated with contrast agents. Any concerning findings on ultrasound should be followed up with a contrast CT scan [3].

Indications

**Figure 2:**
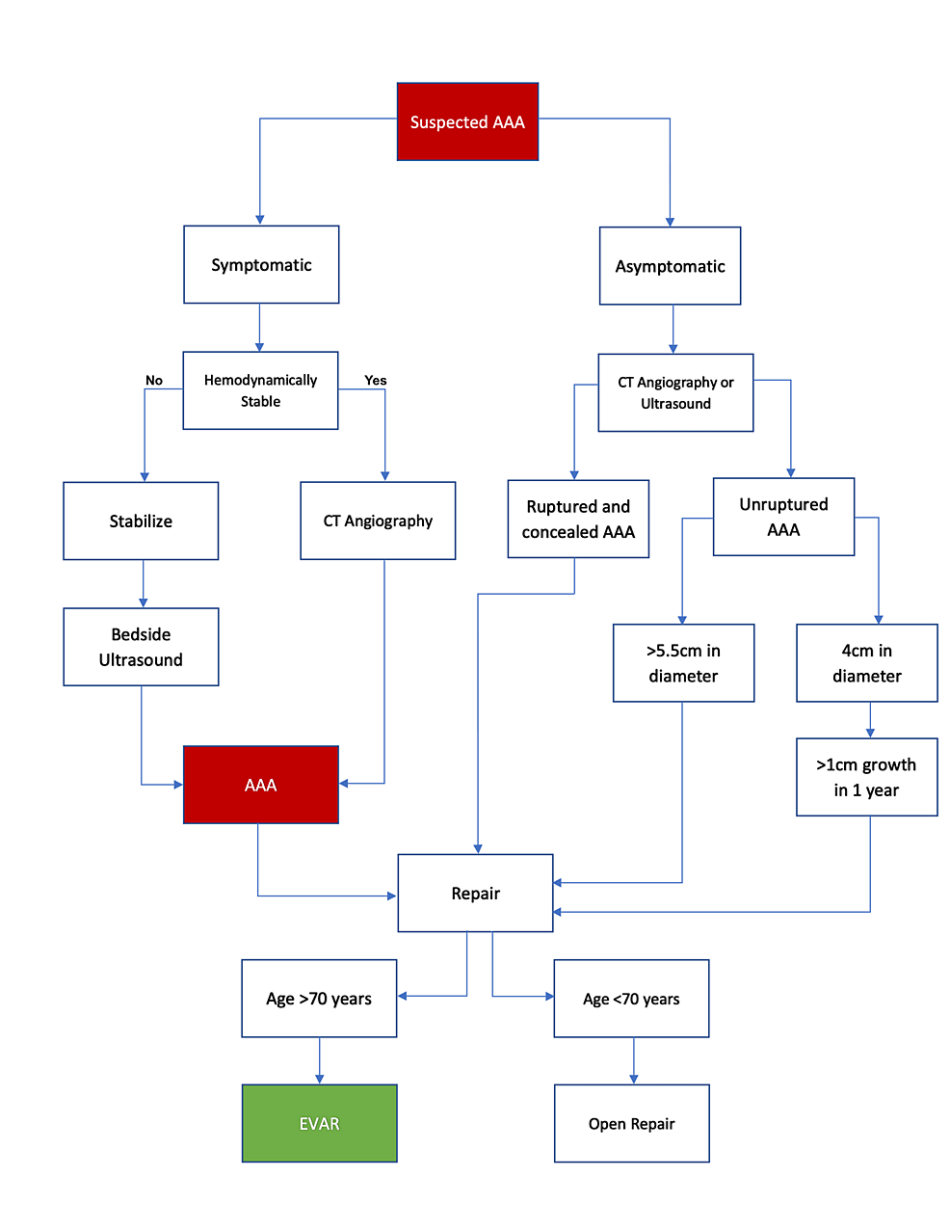
Management protocol for suspected AAAs. Source: [19]. AAA, abdominal aortic aneurysm

According to the NICE Guidelines [19], the management of abdominal aortic aneurysms depends on whether they are ruptured or unruptured (Figure 2).

Unruptured aneurysms are to be surgically repaired if they are:

i) symptomatic,

ii) larger than 4 cm and with a growth >1 cm in a year, or

iii) asymptomatic and >5.5 cm.

EVAR is preferred in unruptured aneurysms for patients who have anesthetic risks, significant comorbidities, or coexisting abdominal pathologies, such as horseshoe kidney or hostile abdomen [19]. Ruptured aneurysms are a surgical emergency and must be treated immediately by open repair or EVAR. The procedure selection usually depends on the patient's age, with open surgery preferred for patients under 70 years, while EVAR is preferred for patients over 70 years [19]. A large-scale meta-analysis of four multicenter randomized clinical trials of EVAR versus open repair conducted by Powell et al. [21] in 2017 analyzed 2,783 patients, and their results showed that EVAR had an early survival advantage over open repair, and the 30-day operative mortality was significantly less in EVAR (16 deaths versus 40 deaths).

Complications

**Figure 3:**
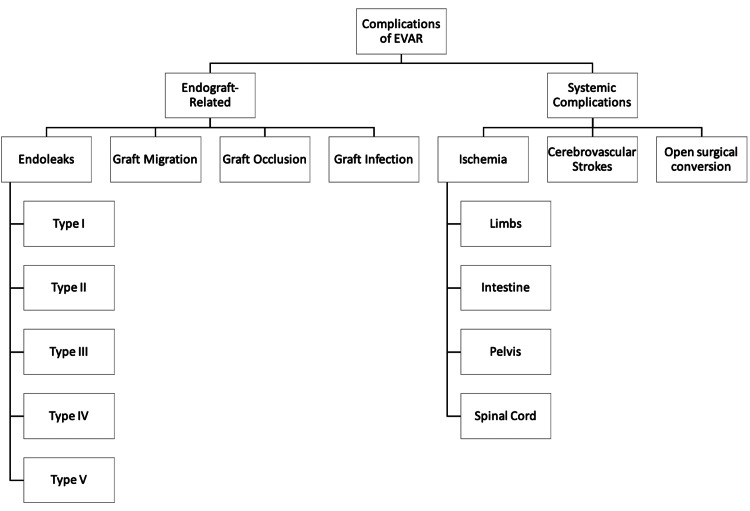
Complications of EVAR. Figure credits: Vishnu R. Yanamaladoddi. EVAR, endovascular aneurysm repair

There are several complications associated with EVAR. These can be grouped into endograft-associated complications and systemic complications (Figure 3). 

Endograft-related complications include endoleaks (15%-30% of cases) [22], graft migration (1%-10% of cases) [22], occlusion of endograft limbs (2%-4% of cases) [22], and infection of the graft (0.6%-3.0% of cases) [23].

Systemic complications include ischemia of the limbs, pelvis, colon, and spinal cord (9% of cases) [24], cerebrovascular strokes (4%-8% of cases) [22], and open surgical conversion (0.6%-4.5% of cases) [22].

Endoleaks

**Figure 4:**
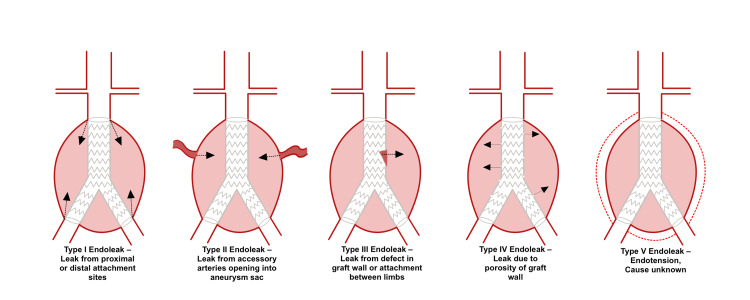
Types of endoleaks. Figure credits: Vishnu R. Yanamaladoddi.

Endoleaks are the most common complication post-EVAR and are seen in up to 30% of cases. Endoleaks indicate blood flow into the aneurysm sac, outside of the graft, and after repair and are thus indicative of treatment failure [25]. Endoleaks are classified based on the site of origin (Figure 4). Type I involves the proximal (Ia) or distal (Ib) graft attachment sites. Type II leaks involve retrograde from branches of the aorta such as the inferior mesenteric artery (IMA). Type III leaks are caused by the discontinuity in the fabric of the graft, either due to tearing or irregularity in the overlap. Type IV leaks are due to porosity in the wall of the graft. Type V leaks are those in which the aneurysm sac is growing with no identifiable leaks [26].

Type I Endoleaks

Type I endoleaks (T1Es) occur due to insufficient graft closure at the endograft proximal (Ia) and distal (Ib) attachment sites. Faries et al. [27] found that T1E occurs in nearly 9% of cases who underwent EVAR. A study by Tan et al. [28] found that among 2,402 EVARs who had unruptured AAA in their sample group, 80 patients (3.3% of the group) had T1E. The study also revealed several factors that predisposed patients to T1E, including female gender, age > 70 years, graft diameter > 3 cm, and unplanned graft extension during surgery, with odds ratios at 2.2, 2.0, 2.6, and 4.6, respectively.

T1Es are a serious complication and may lead to the expansion of the aneurysm sac and its eventual rupture [29]. They must be monitored and treated based on the progression. A retrospective study conducted by Matsumura and Moore [30] in 1998 found that out of 28 patients who had leaks (six proximal, 14 distal, three proximal and distal, and five indeterminate), 14 patients had their leaks sealed spontaneously. Another retrospective study conducted by Kim et al. [31] in 2013 identified similar results, finding that T1E spontaneously closed in seven out of 10 patients. The other patients required treatment to correct the defect. A study by Millen et al. [32] in 2015 found that in 44 patients with T1E, 33 had persistent endoleaks, of which 31 had spontaneous resolution (94%), results that were in tandem with previous studies. Tan et al. [28] had similar results in an observational study with a 90% spontaneous resolution rate. These studies have been summarized in Table 1. 

**Table 1:** Outcomes of type I endoleaks. T1E, type I endoleak

Study	Design	Population	Cases of T1E	Results
Matsumura et al. (1998) [30]	Retrospective study	68 patients in the United States who underwent repair between 1993 and 1995	28	14 patients had spontaneous resolution
Kim et al. (2014) [31]	Retrospective study	86 patients in South Korea between 2000 and 2012	10	7 patients had spontaneous resolution
Millen et al. (2015) [32]	Retrospective study	209 patients in the United Kingdom between 2011 and 2013	33	31 patients had spontaneous resolution
Tan et al. (2016) [28]	Retrospective study	2402 patients in the Vascular Study Group of New England from 2002.	47	44 patients had spontaneous resolution

Treatment options for persistent type Ia endoleaks include balloon expansion, stent insertion, proximal cuff insertion, chimney EVAR (chEVAR), fenestrated EVAR (fEVAR), and the use of anchors and embolization. Type Ib endoleaks are easier to treat and are usually treated by extension of the endograft distally. In case these methods fail, open surgical conversion may be necessary to correct the endoleak [33]. Studies about the various treatment modalities for T1E have been listed in Table 2. Each procedure has a high technical success rate, ranging between 89.7% and 100%. AbuRahma et al. [34] conducted a study in 71 cases of type Ia endoleak management with a Palmaz/Proximal cuff and found a technical success of 100%. Lu et al. [35] found a high success rate in trials of embolization with fibrin glue in 42 cases. EndoAnchors have been proven to be quite useful as well, as evidenced in a study by Jordan et al. [36] in 2014 where he found a technical success rate of 94% in 66 cases.

**Table 2:** Various procedures to treat T1Es and their outcomes. T1E, type I endoleak; chEVAR, chimney endovascular aneurysm repair; fEVAR, fenestrated EVAR

Subgroup of endoleaks	Reference	Procedure	Cases of T1E	Results
Type Ia	Lu et al. (2010) [35]	Embolization (fibrin glue sac)	42	98% technical success
	Katsargyris et al. (2013) [37]	fEVAR	26	92.3% technical success
	Jordan et al. (2014) [36]	EndoAnchors	66	94% technical success rate
	AbuRahma et al. (2017) [34]	Palmaz/Proximal Cuff	71	100% technical success
	Ronchey et al. (2018) [38]	chEVAR	39	89.7% technical success
Type Ib	Massoni et al. (2018) [39]	Distal extension of the endograft	29 patients	100% technical success

Type II Endoleaks

Type II endoleaks (T2Es) are due to the flow of blood from collateral vessels opening into the aorta. They are the most common subtype of endoleaks. The associated collateral vessels in question are usually the lumbar artery and the IMA. A systematic review conducted by Sidloff et al. [40] covering 21,744 patients showed that T2E occurred in 1,515 patients, with an incidence of 10.2%. Similar results were found when van Marrewijk et al. [41] conducted a retrospective study with a cohort of 3,595 patients and found 320 patients had a T2E, an incidence of 9%.

They are relatively benign and usually self-resolve within six months. Table 3 summarizes the various studies on the outcomes of T2E. Makaroun et al. [42] conducted a follow-up study of 20 patients with T2E in 1999 and found that nine had spontaneously resolved by six months (56.25%). A similar study conducted by Silverberg et al. [43] in 2006 with a cohort of 154 patients found that 75% of T2E closed spontaneously within a five-year period, which was substantially more than the findings of the previous study. However a larger scale study in T2Es persisting longer than six months conducted by van Marrewijk et al. [41] identified sac enlargement in 55% of cases.

**Table 3:** Summary of outcomes of T2Es. T2E, type II endoleak

Outcome	Study	Cases of T2E	Results
Sac enlargement	van Marrewijk et al. (2002) [41]	3,595 patients	55% of patients showed aneurysm growth within three years
Spontaneous closure of T2E	Makaroun et al. (1999) [42]	20 patients	56.25% of cases showed spontaneous resolution
	Silverberg et al. (2006) [43]	154 patients	75% of cases showed spontaneous resolution

T2Es are usually treated when they cause an aneurysm growth of >5 mm over six months [44]. There are various methods to treat T2Es listed in Table 4, and they involve the disjunction of the accessory branches at their conjecture with the aneurysm. The endovascular options include transarterial embolization, translumbar/direct sac puncture embolization, and transcaval embolization (Table 4). Surgical treatment modalities include ligation of the feeder arteries or conversion to open repair [45]. Studies have found widely varying results regarding the success rates of transarterial embolization. Funaki et al. [46] conducted a retrospective review and found a technical success rate of 88% in treating T2E. However, cohort studies conducted by Müller-Wille et al. [47] and Haq et al. [48] found much lower success rates at 54.5% and 29.4%, respectively. Ribé et al. [49], however, found a higher success rate of 100%, in support of Funaki et al. [46], in a retrospective review conducted in 2017. Khaja et al. [50] conducted a cohort study in 2014 on translumbar embolization and found a technical success rate of 84.6%. Results supporting this study were found by Carrafielo et al. [51] in 2016, with technical success rates of 100%. However, a cohort study by Rahimi et al. [52] in 2018 found a much lower success rate of 48.2%. Transcaval embolization is another procedure used to treat T2E. Gandini et al. [53] in a cohort study found a technical success rate of 100%. A study by Giles et al. [54] in 2015 found a high technical success rate of 83%.

**Table 4:** Various procedures used for treating T2E and their success rates. T2E, type II endoleak

Procedure	Reference	Type of study	Patients with T2E	Results
Transarterial embolization	Funaki et al. (2012) [46]	Retrospective review	16	88% technical success
	Müller-Wille et al. (2013) [47]	Cohort study	11	54.5% technical success
	Haq et al. (2017) [48]	Cohort study	37	29.4% technical success
	Ribé et al. (2017) [49]	Retrospective review	18	100% technical success
Translumbar embolization	Khaja et al. (2014) [50]	Cohort study	13	84.6% technical success
	Carrafiello et al. (2016) [51]	Retrospective review	8	100% technical success
	Rahimi et al. (2018) [52]	Cohort study	29	48.2% technical success
Transcaval embolization	Gandini et al. (2014) [53]	Cohort study	29	100% technical success
	Giles et al. (2015) [54]	Retrospective review	29	83% technical success

Type III Endoleaks

Type III endoleaks (T3Es) are of two types. Type IIIa endoleaks involve discontinuity between components of the graft, while type IIIb endoleaks involve defects in the graft body itself [55]. In a retrospective study conducted by Maleux et al. [56], in 965 cases who underwent endovascular repair, 25 (2.1%) patients were identified with T3Es. Of the 25 patients identified with T3Es, type IIIa endoleaks were found in 56% (14/25) and type IIIb in 44% (11/25). In the Veterans Affairs Open Versus Endovascular Repair (OVER) Trial of the Abdominal Aortic Aneurysms study, a randomized controlled trial conducted by Lal et al. [57], it was found that out of 187 endoleaks, 3% were of type III.

T3Es are associated with a high risk of rupture, on par with that of T1E, and must be monitored and treated appropriately [58]. Treatment of T3E is via endovascular repair. By deploying additional graft pieces and creating a bridge to cover the disconnected components, these leaks are relined effectively, creating a new graft [55].

Type IV Endoleaks

Type IV endoleaks (T4Es) involve the flow of blood into the aneurysm sac due to the increased porosity in the graft material. Lal et al. [57] found T4E in 3% of their study population (187). Espinosa et al. [59] found the prevalence of T4E at 0.3% in their study population of 193 patients who underwent EVAR. This type of endoleak is usually transient and halts after the completion of postoperative prophylactic therapy [20]. T4Es are rare in newer devices and usually do not require further intervention [45].​​​​​​

Type V Endoleaks

Type V endoleaks (T5Es) are also referred to as endotension and are a diagnosis of exclusion. There is an increase in aneurysm sac size post EVAR without an identifiable endoleak. Lal et al. [57] found T5E in 6% of their study population. Turney et al. [60] found similar data in their comparative study, with 6% of patients presenting with T5E/endotension. T5Es require treatment when there is sac growth >1 cm. The treatment involves relining of the graft or conversion to open repair [45].

Limitations

This study has a few limitations. The study describes the type of endoleaks and the various treatment modalities for each of them; however, it does not go in depth to describe each treatment individually. Several studies also have limited sample sizes. There is also a paucity of data regarding T4Es and T5Es.

## Conclusions

This study delves into the discussion of abdominal aortic aneurysms and their endovascular repair. EVAR serves as a valuable treatment option for AAAs and has better operative outcomes and reduced morbidity as compared to open repair, but they are associated with a major complication of endoleaks. Endoleaks are associated with sac enlargement and ruptures and lead to increased rates of re-intervention and death. This study helps to elaborate on the five subgroups of endoleaks and their incidence, progression, and various treatment modalities associated with them. A brief overview of the procedure of EVAR was given, and the development of endoleaks was also discussed elaborately. Clinically, this may serve to raise awareness of the most common complication associated with EVAR and how to promptly identify and proceed with their management. We believe this study can be used for further research into the various endoleaks and how to avoid them to minimize their impact on postoperative mortality following the EVAR of AAAs. We believe that further studies should be conducted into preoperative prevention and faster identification of endoleaks, and subtype-specific protocols of management should be developed to optimize patient outcomes.
